# Flower bud proteome reveals modulation of sex-biased proteins potentially associated with sex expression and modification in dioecious *Coccinia grandis*

**DOI:** 10.1186/s12870-019-1937-1

**Published:** 2019-07-23

**Authors:** Ravi Suresh Devani, Tejas Chirmade, Sangram Sinha, Abdelhafid Bendahmane, Bhushan B. Dholakia, Anjan Kumar Banerjee, Jayeeta Banerjee

**Affiliations:** 1 Biology Division, Indian Institute of Science Education and Research (IISER), Pune, 411008 India; 2 Biochemical Science Division National Chemical laboratory (CSIR-NCL), Pune, 411008 India; 3 Academy of Scientific and Innovative Research (AcSIR), New Delhi, India; 4 Department of Botany, Tripura University, Suryamaninagar, Tripura 799022 India; 5 IPS2, INRA, CNRS, University Paris Sud, University of Evry, University of Paris Diderot, University of Paris Saclay, Batiment 630, 91405 Orsay, France; 6 Department of Molecular Biology & Bioinformatics, Tripura University, Suryamaninagar, Tripura 799022 India

**Keywords:** *Coccinia grandis*, Dioecy, Sex modification, Stamen arrest, Pollen fertility, Proteomics

## Abstract

**Background:**

Dioecy is an important sexual system wherein, male and female flowers are borne on separate unisexual plants. Knowledge of sex-related differences can enhance our understanding in molecular and developmental processes leading to unisexual flower development. *Coccinia grandis* is a dioecious species belonging to Cucurbitaceae, a family well-known for diverse sexual forms. Male and female plants have 22A + XY and 22A + XX chromosomes, respectively. Previously, we have reported a gynomonoecious form (22A + XX) of *C. grandis* bearing morphologically hermaphrodite flowers (GyM-H) and female flowers (GyM-F). Also, we have showed that foliar spray of AgNO_3_ on female plant induces morphologically hermaphrodite bud development (Ag-H) despite the absence of Y-chromosome.

**Results:**

To identify sex-related differences, total proteomes from male, female, GyM-H and Ag-H flower buds at early and middle stages of development were analysed by label-free proteomics. Protein search against the cucumber protein sequences (Phytozome) as well as in silico translated *C. grandis* flower bud transcriptome database, resulted in the identification of 2426 and 3385 proteins (FDR ≤ 1%), respectively. The latter database was chosen for further analysis as it led to the detection of higher number of proteins. Identified proteins were annotated using BLAST2GO pipeline. SWATH-MS-based comparative abundance analysis between Female_Early_vs_Male_Early, Ag_Early_vs_Female_Early, GyM-H_Middle_vs_Male_Middle and Ag_Middle_vs_ Male_Middle led to the identification of 650, 1108, 905 and 805 differentially expressed proteins, respectively, at fold change ≥1.5 and *P* ≤ 0.05. Ethylene biosynthesis-related candidates as highlighted in protein interaction network were upregulated in female buds compared to male buds. AgNO_3_ treatment on female plant induced proteins related to pollen development in Ag-H buds. Additionally, a few proteins governing pollen germination and tube growth were highly enriched in male buds compared to Ag-H and GyM-H buds.

**Conclusion:**

Overall, current proteomic analysis provides insights in the identification of key proteins governing dioecy and unisexual flower development in cucurbitaceae, the second largest horticultural family in terms of economic importance. Also, our results suggest that the ethylene-mediated stamen inhibition might be conserved in dioecious *C. grandis* similar to its monoecious cucurbit relatives. Further, male-biased proteins associated with pollen germination and tube growth identified here can help in understanding pollen fertility.

**Electronic supplementary material:**

The online version of this article (10.1186/s12870-019-1937-1) contains supplementary material, which is available to authorized users.

## Background

Flowering plants show three major sexual systems viz. hermaphroditism, monoecy and dioecy. Around 90% of the angiosperm species are hermaphroditic bearing perfect flowers having both male as well as female reproductive sex organs [1]. Monoecy exists at a frequency of ~ 5% in angiosperms, wherein unisexual male and female flowers are produced on the same individual plant. Remaining ~ 5% angiosperm species are dioecious, having separate unisexual plants bearing either only male or female flowers [2–4]. Dioecious species show patchy phylogenetic distribution and are reported in around three-fourth of the angiosperm families. This indicates that the evolution of dioecy has occurred multiple times in different families independently and hence, the molecular mechanisms of sex determination might vary between distant dioecious species and is a matter of great research interest [5–7]. Out of ~ 14,600 known dioecious species in 200 families, plant sex chromosomes have been reported in just around 40 species till date [8]. The mechanism of sex determination in plants can be complex and it may also involve environmental factors apart from genetic factors [9].

*Coccinia grandis* (L.) Voigt, is a dioecious member of Cucurbitaceae, a family known for its diverse sexual systems [10]. In general, *C. grandis* is not widely used as a model system to understand sex expression and modification. Commonly known as ivy gourd, *C. grandis* is also used as a vegetable. Male and female unisexual flowers are borne on separate plants. Similar to *Silene latifolia* (Caryophyllaceae), the presence of large Y-chromosome in male plant determines the sex [11–13]. The chromosome constitution of male and female *C. grandis* plants found to be 22A + XY and 22A + XX, respectively [14–16]. The male flower is characterized by the presence of three convoluted (bithecous) stamens and it has no carpel, whereas the female flower possess three rudimentary stamens that surround the three fused carpels having an inferior ovary [17, 18].

Two possible explanations can be put forward with respect to unisexual flower development. (1) Primordia for both male and female sex organs are initiated during the early stages of flower development and one of them gets aborted during the later stages (eg: *S. latifolia*). (2) The flower buds are unisexual right from the inception with the primordia initiation for only one of the two sex organs (eg: *Thalictrum dioicum*) [19, 20]. In some species, inappropriate sex organs are retained in the rudimentary form instead of getting aborted (eg: *Rumex acetosa*) [21]. Previously, we have demonstrated that application of AgNO_3_ on female *C. grandis* plant modifies the sex expression by inducing stamen development leading to formation of hermaphrodite flowers (such flowers will be referred to as Ag-H hereafter) [18]. Ag^+^ ion is a known inhibitor of ethylene response [22]. Binding of Ag^+^ ion to the ethylene receptor locks the conformation such that it remains continuously active and represses the ethylene responses [23]. Silver compounds (AgNO_3_ and Ag_2_S_2_O_3_) have masculinizing effect on monoecious plants (*Cucumis sativus*) as well as female plants of dioecious species (*S. latifolia* and *Cannabis sativa*) [24–26]. However, the molecular mechanism of AgNO_3_-mediated induction of stamen development remains unknown till date [26] .

At present, our knowledge about the sex determination mechanisms in plants is fairly limited. Major limitation for studying mechanisms of sex determination in plants is that majority of the dioecious plants are non-model organisms without the availability of genome sequence. Hence, the rate at which sex-linked genes are identified from dioecious plant species is very low [27]. However, the advent of NGS (next-generation sequencing) technologies has enabled the high-throughput identification of sex-biased genes from dioecious plant species in recent times [27]. Also, advanced proteomic approaches may lead to the identification of novel sex-linked proteins that can eventually expand our understanding in evolutionary, developmental and molecular mechanism(s) associated with sex determination and modification.

Muyle*,* et al. [27] took an RNA-Seq approach to identify sex-biased gene expression in *S. latifolia* and demonstrated the dosage compensation in plants for the first time. Similarly, other transcriptome studies in this plant have helped in understanding the Y chromosome degeneration and identification of new sex-linked genes [28, 29]. Another study in persimmon (*Diospyros lotus*) showed that *OGI* (*Oppressor of MeGI*), a Y-chromosome-encoded small RNA governs pollen fertility by targeting a homeodomain transcription factor *MeGI* in a dose-dependent manner [30]. Comparative de novo transcriptomics approach taken in *Asparagus* resulted in identification of genes involved in pollen microspore and tapetum development that were expressed specifically in male flowers [31]. Similar transcriptomics studies have also been carried out in papaya and cucumber that has led to better understanding of sex determination [32–34]. It can be noted that recently, we have carried out a de novo transcriptome profiling from male, female, GyM-H and Ag-H flower buds of *C. grandis* and identified many sex-biased genes that can provide crucial insights in to stamen arrest, pollen fertility and sex modification mechanism [35]*.*

By using transcriptomics over genomics, it is possible to capture differential gene regulations that may arise due to changes in environmental cues/signals or based on the sex of the plant as in case of dioecious species. However, transcriptomics has a disadvantage that the mRNA abundance may not always accurately reflect protein levels, which are the end-products in the realization of hereditary information carrying out various structural and functional duties [36, 37]. Moreover, only proteomics can capture the post-translational modifications, which are very well-known to control protein functions. However, proteomics study generally depends on the availability of genomic/transcriptomic data, which is limited for most of the dioecious species. Hence, there are only few studies, which have employed proteomics approach in order to understand sex determination and differentiation in dioecious plants [38]. One such study carried out in *Asparagus* identified differentially accumulated proteins in the form of spots on 2-D gels from flowers of male and female plants [39]. Another study in *Pistacia vera* purified a 27 kDa glycoprotein specific to female inflorescence [40]. Proteomic approaches has also been attempted to identify sex-linked proteins in *Ginkgo biloba*. A 28 kDa protein specific to male and 36 kDa as well as 92 kDa proteins specific to female inflorescences have been identified [38]. Recently, Manzano*,* et al. [41] showed that overexpression of aerolysin-like protein from the dioecious plant *R. acetosa* induces male sterility in transgenic tobacco.

In order to decipher differences in protein abundances among flower buds of male, female, gynomonoecious and chemically masculinized female plants of *C. grandis*, total proteome profiling was carried out at early and middle stages of flower development. Proteins were identified using IDA (Information dependent acquisition)-derived data using in silico translated in-house *C. grandis* flower bud transcriptome [35] database followed by label-free quantification using SWATH-MS (Sequential Window Acquisition of All Theoretical Mass Spectra) analysis. Differential protein abundance among various sex forms of *C. grandis* was studied to identify players potentially involved in sex expression and modification. Our study has identified key proteins potentially involved in the processes of stamen arrest, AgNO_3_-mediated sex modification and pollen fertility in dioecious *C. grandis*.

## Results

### Flower bud proteome of *C. grandis*

**Fig. 1:**
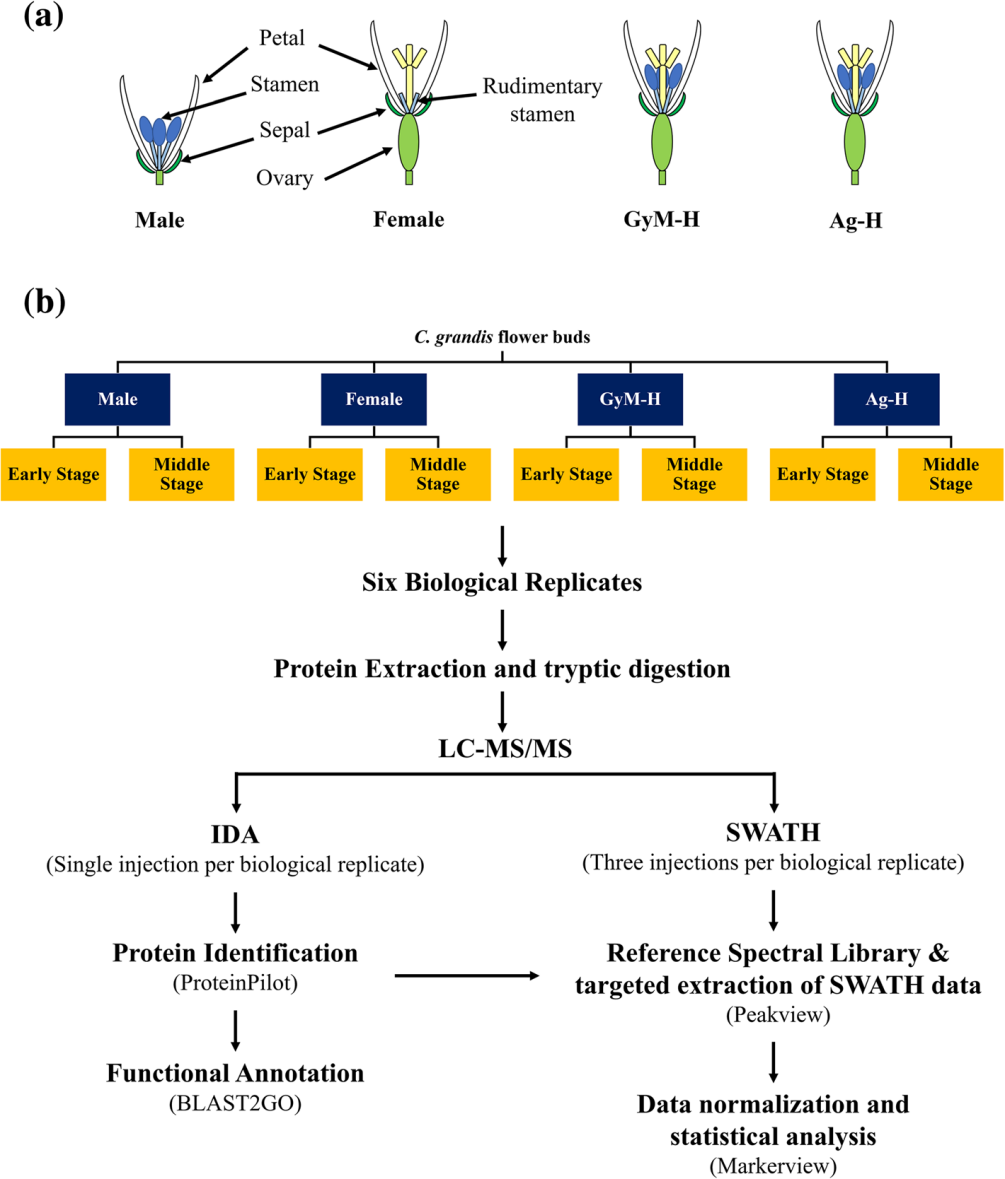
Scheme for *Coccinia grandis* flower bud proteomics. **a** Schematic representation of *C. grandis* flower types used in current study. **b** Experimental procedure for flower bud label-free proteomics

The tryptic-digested protein samples were analysed by tandem LC-MS in IDA model, and the acquired data was processed by Paragon TM as per experimental scheme shown in Fig. 1. A total of 1193777 spectra (68.8% of total spectra) corresponding to 66842 distinct peptides representing total 3387 proteins (Additional file 1: Data S1) were identified from different categories of *C. grandis* flower buds with FDR ≤ 1% (Unused ProtScore Cutoff > 2.0) using in silico translated in-house *C. grandis* flower bud transcriptome (Table 1). Simultaneously, protein identification using *Cucumis sativus* protein sequences as database led to the identification of 2426 proteins, out of which 434 were new proteins that were not detected using the in silico translated in-house *C. grandis* flower bud transcriptome (Additional file 1: Data S1). This has further improved the number of proteins identified from the *C. grandis* flower buds in the present study. However, all the following analyses were carried out using *C. grandis* transcriptome as the database, since it led to the detection of a higher number of proteins.

**Table 1:** Summary of protein identification with AB SCIEX Triple TOF 5600 platform using Paragon Algorithm with ProteinPilot v5.0.1

Unused (Conf) Cutoff	Proteins Detected	Proteins Before Grouping	Distinct Peptides	Spectra Identified	% Total Spectra
> 2.0 (99)	3387	14816	66842	1193777	68.8
> 1.3 (95)	3855	17469	68548	1205044	69.5
> 0.47 (66)	4649	21946	71945	1217799	70.2
Cutoff Applied: > 0.05 (10%)	8979	59421	87507	1256111	72.4

### Annotation of identified proteins

**Fig. 2:**
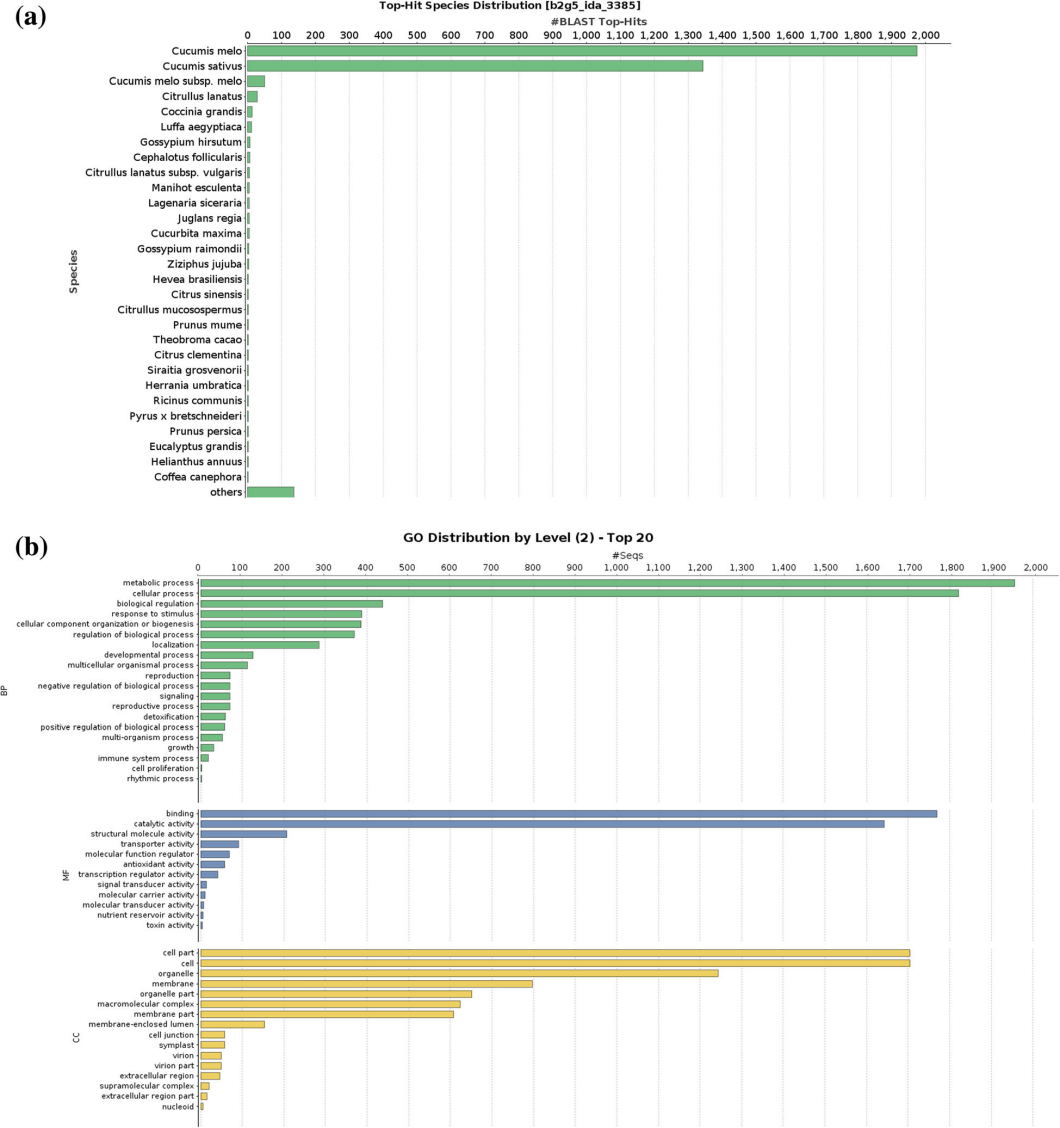
Annotation of *C. grandis* flower bud proteome with BLAST2GO v5. **a** BLAST Top-Hit species distribution when compared with nr database using blastp (**b**) GO category (level 2) distribution of *C. grandis* flower bud proteome

Proteins identified using ProteinPilot software were searched against viridiplantae subset of nr (non-redundant proteins) database using BLASTp with an e-value threshold of 1e-3 (Fig. 2a). Total 3377 proteins fetched at least one BLAST hit. More than 95% of the proteins gave top BLAST hit against *C. melo* (melon) or *C. sativus* (cucumber). BLAST2GO was used to annotate and classify the identified proteins into three major gene ontology categories (molecular function, cellular component and biological process) (Additional file 2: Data S2). For biological process, majority of the proteins were involved in metabolic process (1956 proteins) and cellular process (1821 proteins) (Fig. 2b). Interestingly, we could detect 71 proteins annotated with reproduction (GO:0000003) term (Fig. 2b). Binding (1769 proteins) and catalytic activity (1642 proteins) were two major categories for molecular function (Fig. 2b). Also, 41 proteins with transcription regulator activity were detected (Fig. 2b). Among cellular component, 1243 proteins associated with organelle, while 797 were related with membrane (Fig. 2b) function. The most common enzymes were hydrolases, transferases and oxidoreductases in the identified proteome based on enzyme code distribution analysis (Additional file 3: Figure S1).

### Differentially expressed proteins involved in the flower development

**Fig. 3:**
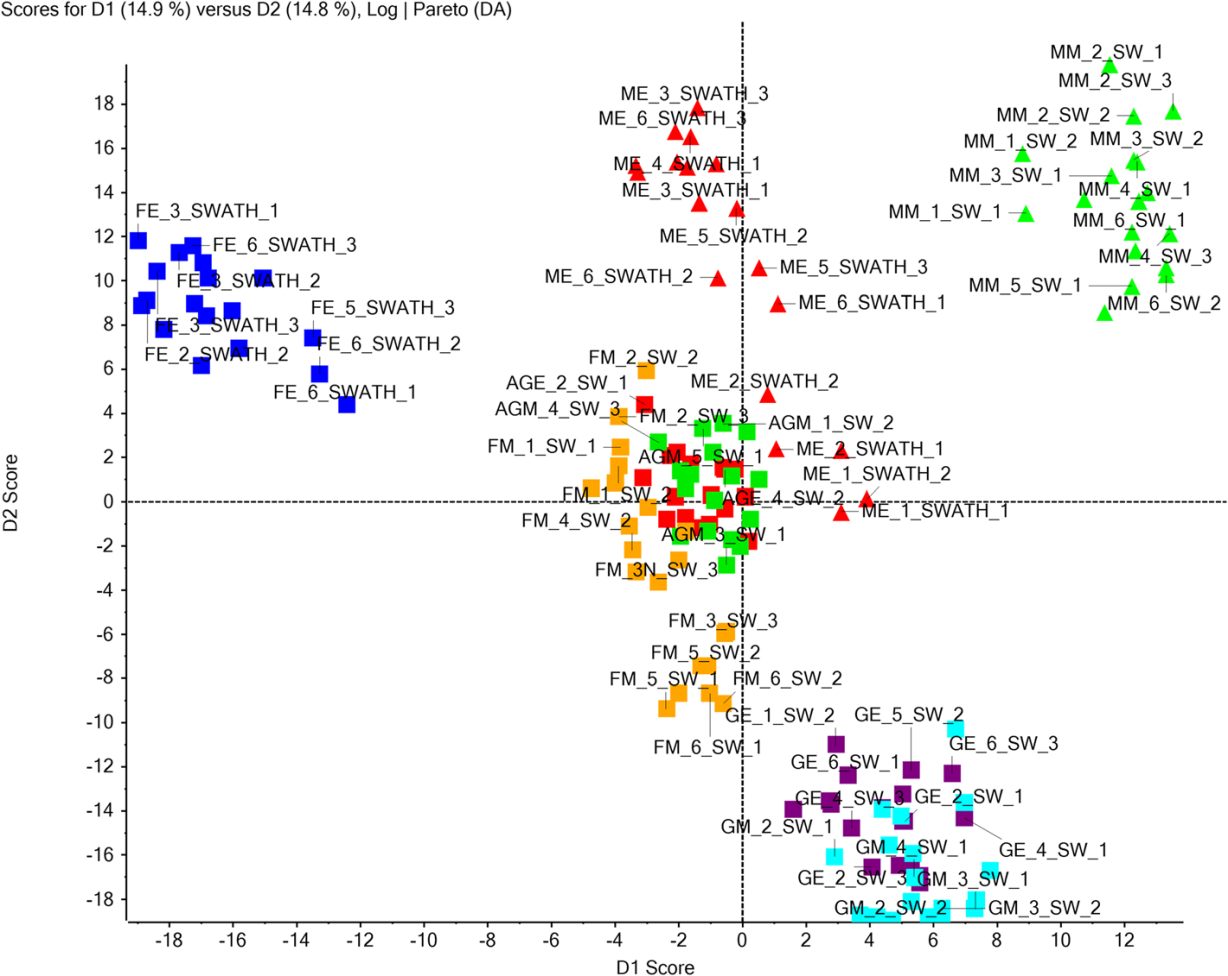
PCA-DA analysis showing relationship between all the *C. grandis* flower bud samples as well as replicates in the study. AGE (Red square), Early-staged Ag-H; FE (Blue square), Early-staged Female; GE (Purple square), Early-staged GyM-H; GM (Turquoise square), Middle-staged GyM-H; ME (Red triangle), Early-staged Male; MM (Green triangle), Middle-staged Male; AGM (Green square), Middle-staged Ag-H; FM (Orange square), Middle-staged Female

**Fig. 4:**
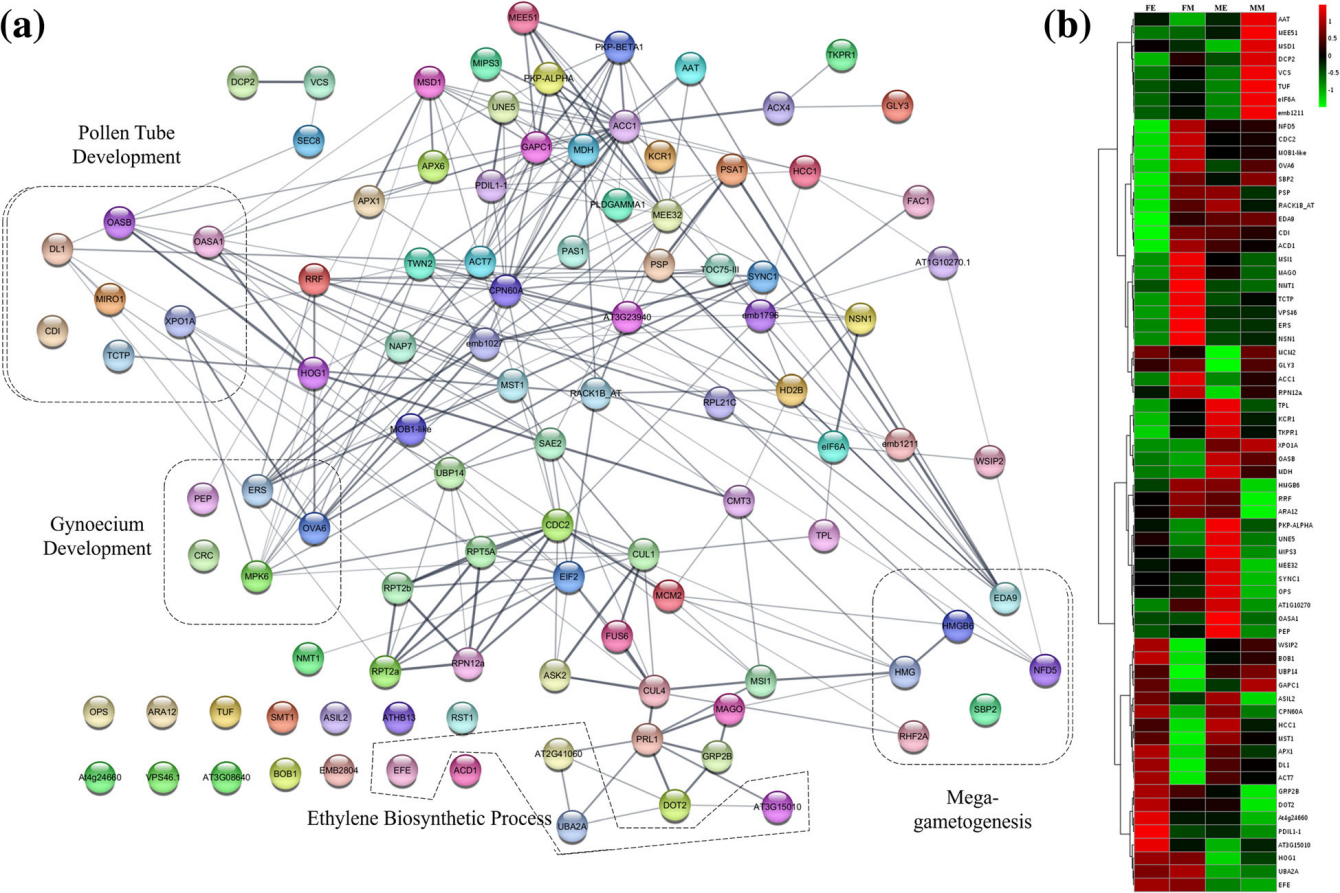
Functional interactions and abundance profiles of proteins involved in female reproductive organ, embryo and seed development. **a** Protein-protein functional interaction network generated using STRING. **b** Hierarchical clustering heatmap showing expression profiles for the selected proteins. Thickness of the line in interaction maps indicate the degree of confidence for prediction of the interaction. Dotted boxes with labels highlight the subset of proteins involved in respective processes. Z-score or scaled expression value of each protein is plotted in red-green color scale. The red color of the tile indicates higher level and green indicates lower level. FE (Red), Early-staged Female; FM (Green), Middle-staged Female; ME (Blue), Early-staged Male; MM (Turquoise), Middle-staged Male

**Fig. 5:**
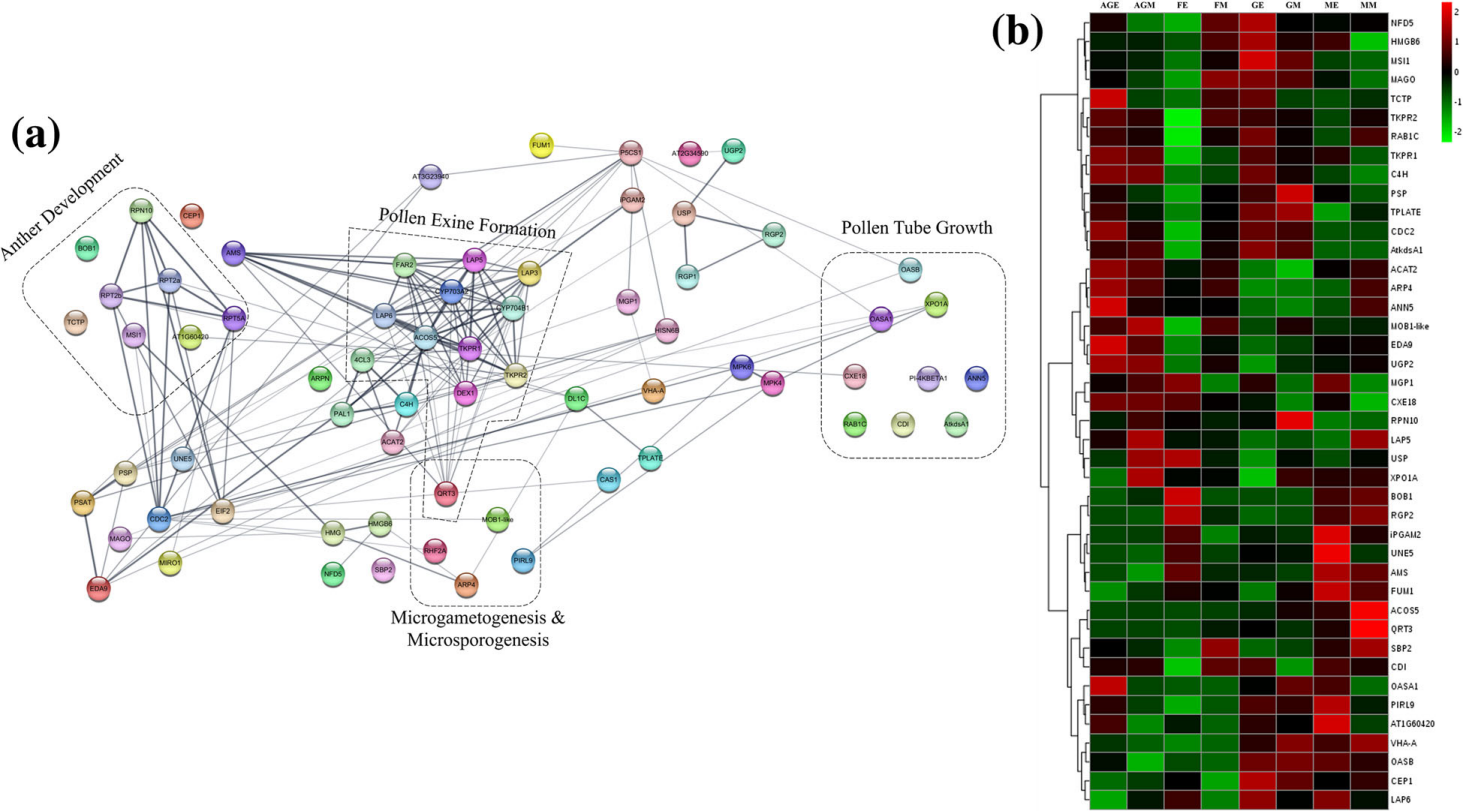
Functional interactions and abundance profiles of proteins involved in male reproductive organ development. **a** Protein-protein functional interaction network generated using STRING. **b** Hierarchical clustering heatmap showing expression profiles for the selected proteins. Thickness of the line in interaction maps indicate the degree of confidence for prediction of the interaction. Dotted boxes with labels highlight the subset of proteins involved in respective processes. Z-score or scaled expression value of each protein is plotted in red-green color scale. The red color of the tile indicates higher level and green indicates lower level. AGE (Red), Early-staged Ag-H; FE (Blue), Early-staged Female; GE (Pink), Early-staged GyM-H; GM (Yellow), Middle-staged GyM-H; ME (Grey), Early-staged Male; MM (Black), Middle-staged Male; AGM (Green), Middle-staged Ag-H; FM (Turquoise), Middle-staged Female

In order to understand how the expression of proteins was regulated during the development of male flower in contrast to female flower, quantitative proteomics was performed using label-free SWATH-MS to identify the DEPs (differentially expressed proteins). Based on the identified proteins from all samples, SWATH-MS analysis led to the detection of total 2262 proteins that were quantified from 144 SWATH data files. PCA-DA (Principal Component Analysis and Discriminant Analysis) showed that replicates belonging to each category of the samples were fairly clustered together. Additionally, upon PCA-DA analysis, the relationship or differences between different samples as per tissue and/or developmental stages was noticeable. For example, female_early and male_middle flower buds were divergent compared to the rest of flower types (Fig. 3). This clearly reflects the significant variations in identified proteins as well as their expression levels among different tissues and their developmental stages (Additional file 4: Figure S2). All pairwise comparisons were then made to identify DEPs at fold change ≥1.5 and *P* ≤ 0.05. Pairwise comparisons between the proteome profiles of different flower buds at early and middle stages of development are summarized in Table 2. Female_early vs. male_early comparison was made in order to identify key players regulating stamen arrest during female bud development. Interestingly, proteins involved in ethylene biosynthesis such as UBA2A (UBP1-associated protein 2A) (1.61-fold), UBA2C (UBP1-associated protein 2C) (2.1-fold) and EFE (Ethylene-forming enzyme) (1.64-fold) were upregulated in early-staged female buds compared to male buds (Fig. 4a & b). Also, ERS (ethylene response sensor) (4.83-fold Middle) was enriched in middle-staged female buds compared to male buds (Fig. 4b) (Additional file 5: Data S3). We observed that the expression of AMS (ABORTED MICROSPORES) (0.46-fold) was downregulated in middle-staged female buds compared to male buds (Fig. 5a, b) (Additional file 5: Data S3). Similarly, to identify the effect of AgNO_3_ treatment on female plant that leads to stamen development, we compared protein profiles of Ag-H_early vs. female_early buds. This comparison resulted in the identification of many male function related proteins that were upregulated upon AgNO_3_ treatment during early stage such as UGP2 (UTP-glucose-1-phosphate uridylyltransferase 2) (2.41-fold), EDA9 (EMBRYO SAC DEVELOPMENT ARREST 9) (2.05-fold), TKPR1 (Tetraketide alpha-pyrone reductase 1) (2.72-fold), C4H (Cinnamic acid 4-hydroxylase) (3.53-fold), TPLATE (1.56-fold), CDC2 (Cell division control protein 2 homolog A) (3.52), ANN5 (Annexin D5) (1.88-fold), RAB1C (Ras-related protein RABD2c) (3.13-fold), OASA1 (O-acetylserine sulfhydrylase 1) (3.56-fold), AtkdsA1 (Putative aldolase-type TIM barrel family protein) (4.05-fold), TCTP (Translationally-controlled tumor protein) (2.52-fold) and ACAT2 (Acetoacetyl-CoA thiolase 2) (2.71-fold) (Fig. 5b) (Additional file 5: Data S3). Also, many other male function-proteins like USP (UDP-sugar pyrophosphorylase) (1.69-fold), RPN10 (Regulatory particle non-ATPase 10) (1.39-fold) and LAP5 (LESS ADHESIVE POLLEN 5) (1.69-fold) were enriched in middle-staged Ag-H buds compared to female buds (Fig. 5b) (Additional file 5: Data S3). The other two comparisons (GyM-H_middle vs. male_middle and Ag-H_middle vs. male_middle) led to the identification of potential targets that may be involved in pollen germination and pollen tube growth. Interestingly, AMS (ABORTED MICROSPORES) (0.24 fold in GyM-H, 0.14 fold in Ag-H), UNE5 (UNFERTILIZED EMBRYO SAC 5) (0.47 fold in Ag-H), iPGAM2 (0.49 fold in GyM-H, 0.46 fold in Ag-H), ACOS5 (Acyl-CoA Synthetase) (0.1 fold in GyM-H, 0.03 fold in Ag-H) and QRT3 (QUARTET 3) (0.14 fold in GyM-H, 0.11 fold in Ag-H) were highly expressed in middle-staged male buds compared to GyM-H and Ag-H buds (Fig. 5b) (Additional file 5: Data S3). Similarly, FUM1 (Fumarate hydratase 1) (0.68 fold in GyM-H, 0.65 fold in Ag-H) was enriched in early-staged male buds compared to GyM-H and Ag-H buds (Fig. 5b) (Additional file 5: Data S3). Further, overlap of DEPs among all the pairwise comparisons were analysed for early and middle stages of development (Additional file 6: Figure S3). Interestingly, 66 proteins were unique to the Ag-H_early vs. female_early comparison (Additional file 7: Data S4). These proteins could be of particular interest for understanding all the changes induced by AgNO_3_ leading to sex modification in female plants. Similarly, analysis for middle-staged buds revealed 47 and 73 DEPs that were unique to Ag-H_middle vs. male_middle and GyM-H_middle vs. male_middle comparisons, respectively. These proteins could serve as potential candidates for future studies to understand the pollen fertility in male buds of dioecious cucurbits (Additional file 8: Data S5).

**Table 2:** Summary of differentially expressed proteins (DEPs) in pairwise comparisons between male, female, GyM-H and Ag-H flower buds at early and middle stages of development

Pairwise comparisons	Purpose	Number of DEPs
Ag-H Early vs. Female Early	To identify the effect of AgNO_3_ treatment on female plants that leads to stamen development	1108
Ag-H Early vs. GyM-H Early	To identify common DEPs involved in stamen arrest compared to female buds	617
Ag-H Early vs. Male Early	Potential targets that may be involved in pollen development	872
Ag-H Middle vs. Female Middle	To identify DEPs involved in Ag^+^-mediated stamen growth	381
Ag-H Middle vs. GyM-H Middle	To identify common DEPs involved in stamen arrest compared to female buds	711
Ag-H Middle vs. Male Middle	Potential targets that may be involved in pollen germination and pollen tube growth	805
Female Early vs. GyM-H Early	To identify DEPs involved in stamen arrest	1120
Female Early vs. Male Early	To identify proteins regulating stamen arrest during female bud development	650
Female Middle vs. GyM-H Middle	To identify DEPs involved in stamen arrest during female bud development	653
Female Middle vs. Male Middle	To identify DEPs involved in stamen arrest and pollen development	812
GyM-H Early vs. Male Early	Potential targets that may be involved in pollen development	887
GyM-H Middle vs. Male Middle	Potential targets that may be involved in pollen germination and pollen tube growth	905

### Transcript abundance analysis

**Fig. 6:**
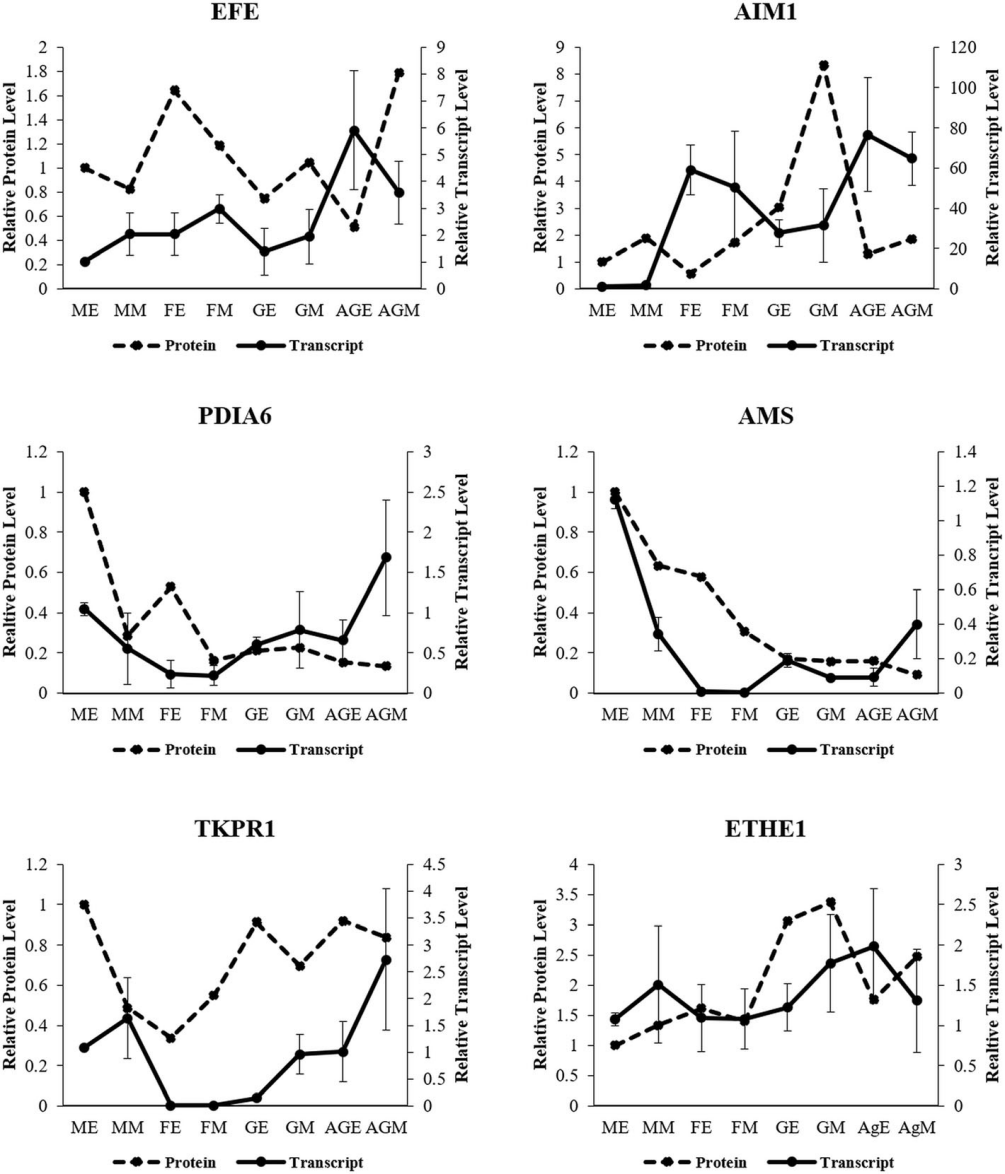
Transcript abundance analysis for selected DE proteins by qRT-PCR with three biological replicates. The relative abundance of transcripts (based on qRT-PCR) and proteins (based on SWATH analysis) in the sample of early-staged male (ME) were set to 1 for plotting the data. Error bars indicate the standard deviation. AgEA, Early-staged Ag-H A; AgEB, Early-staged Ag-H B; FEA, Early-staged Female A; FEB, Early-staged Female B; GEA, Early-staged GyM-H A; GEB, Early-staged GyM-H B; GMA, Middle-staged GyM-H A; GMB, Middle-staged GyM-H B; MEA, Early-staged Male A; MEB, Early-staged Male B; MMA, Middle-staged Male A; MMB, Middle-staged Male B; AgMA, Middle-staged Ag-H A, AgMB, Middle-staged Ag-H B; FMA, Middle-staged Female A; FMB, Middle-staged Female B

Transcript levels of the selected proteins were validated by qRT-PCR analysis using the same tissues. *Coccinia* homologs of *AIM1*, *EFE*, *ETHE1*, *AMS*, *TKPR1* and *PDIA6* showed interesting transcript abundance profiles with respect to flower bud types (Fig. 6). *EFE* showed two-fold expression in early-staged female buds as compared to male buds. Pollen development related transcripts *AMS*, *TKPR1* and *PDIA6* were enriched in male, GyM-H and Ag-H buds compared to female buds. *TKPR1* showed more than 100-fold expression in middle-staged male buds compared to female buds and was enriched in middle-staged GyM-H and Ag-H buds. *AMS* homolog exhibited a 100-fold expression in early-staged male buds compared to female buds; > 5-fold levels compared to GyM-H buds and 10-fold increase compared to Ag-H buds. However, there were marginal differences between the transcript and the protein levels in some of the genes. For example, PDIA6 protein levels were highest in early-staged male buds, while Ag-H buds showed very low levels. However, in contrast to protein abundance, *PDIA6* transcript was most enriched in Ag-H buds. Also, ETHE1 protein levels were found to be the lowest in male bud samples in contrast to the transcript levels.

### Protein interaction network reveals presence of potential targets from reproductive organ development

The proteins identified in relation to reproductive organ, embryo and seed development were further used for protein-protein functional interaction mapping. STRING analysis for 42 male reproductive organ development-related proteins resulted in a functional interaction map indicating anther and pollen development, pollen tube growth as well as microgametogenesis and microsporogenesis related function proteins (Fig. 5a) (Additional file 9: Data S6). Expression analysis of these proteins indicated that majority were enriched in male, GyM-H and Ag-H buds compared to female buds (Fig. 5b). Similarly, another STRING analysis of 66 identified proteins related to female reproductive organ, embryo and seed development led to the identification of protein interaction networks governing gynoecium development, ethylene biosynthesis, megagametogenesis and pollen tube development (Fig. 4a) (Additional file 9: Data S6). Importantly, proteins from gynoecium development and ethylene biosynthesis related clusters were enriched in female buds (Fig. 4b). This might lead to the stamen inhibition in female buds and explain the absence of gynoecium in male buds.

## Discussion

### Comparative profiles of proteins involved in female reproductive functions

Our study is one of the few proteomic reports focused on identification of proteins involved in unisexual flower development. We compared the protein profiles of male and female buds of *Coccinia* at early as well as middle stages of development and identified proteins related to male and female reproductive organ development. At early stage, female flower buds of *C. grandis* exhibit the growth of both stamens and carpels. However, before the flowers reaches to middle stage of development, stamen gets arrested in female buds. The mechanism of stamen arrest has been well studied in monoecious relatives of *Coccinia* such as melon, cucumber and watermelon. However, we have no clue about the stamen arrest in unisexual flower of *Coccinia* [42–45]. ACS (ACC synthase), an enzyme involved in ethylene biosynthesis is found to be expressed at very high levels in female buds of melon, cucumber and watermelon compared to the male buds. High levels of ethylene in female bud act as an inhibitor of stamen development [42–46]. However it is not known, if the ACS function is conserved in dioecious cucurbits. Previously, we have shown that GO terms related to ethylene signalling were enriched in female buds of *C. grandis* [35]. In present study however, we couldn’t detect ACS from the flower buds. Instead, we found that the inducers of ACS, namely UBA2A (UBP1-associated protein 2A) and UBA2C (UBP1-associated protein 2C) had very high abundance in female *C. grandis* buds compared to male buds (Fig. 4a & b). In *Arabidopsis*, Kim*,* et al. [47] showed that overexpression of UBA2A and UBA2C induces the expression of various ACS genes. EFE (Ethylene-forming enzyme), another enzyme involved in ethylene biosynthesis was also enriched in early as well as middle-staged female buds compared to male samples (Fig. 4a, b). *CsACO2* gene, an EFE homolog is known to be essential for the carpel development in cucumber [48]. Recently, Tao*,* et al. [49] reported that Ethylene responsive factor (ERF110) mediates ethylene-regulated transcription of sex determination related orthologous gene *ACS11* in melon and cucumber. This evidence further supports the role of ethylene in governing the sex expression in cucurbits. Moreover, we observed that female buds of *C. grandis* also showed much higher accumulation of ERS (Ethylene response sensor), an ethylene receptor at middle stages of development compared to male buds (Fig. 4a & b) [50]. This indicates that ethylene levels as well as ethylene responses are higher even in the female buds of dioecious *C. grandis* similar to the female buds of monoecious cucurbits. This perhaps explains the cause of stamen arrest in *C. grandis*; however, further functional studies are required to validate this hypothesis. Apart from this, OVA6 (OVULE ABORTION 6) involved in ovule development was enriched in the female buds and the male flowers of *C. grandis* didn’t show any sign of female reproductive organs (Fig. 4a & b) [51]. Levels of proteins involved in embryo development such MAGO, VPS46 (Vacuolar protein-sorting-associated protein 46), PDIL1–1(Protein disulfide isomerase-like 1–1), GRP2B (Glycine-rich protein 2b), At4g24660 (Zinc-finger homeodomain protein 2) were also increased in female buds compared to male counterparts (Fig. 4a & b) [52–55]. MAGO, a protein involved in pollen tube guidance was expressed in female buds at middle stage of development indicating the maturation of female buds for supporting fertilization (Fig. 4a & b) [56].

### AgNO_3_ treatment induces changes in protein profiles affecting sex modification

In previous study, we have shown that foliar spray of AgNO_3_ at optimal concentration (35 mM) can induce stamen development in the newly emerging flower buds of female plants resulting in sex modification [18]. The morphologically hermaphrodite flowers (Ag-H) have full-sized stamens surrounding the carpels. Based on earlier de novo transcriptomics study and the literature available for sex determination mechanism in monoecious relatives of *Coccinia* (melon, cucumber and watermelon), we hypothesized that Ag^+^ ions bring about the sex modification by inhibiting ethylene signalling [35, 42–45, 48, 57, 58]. *CmACS7* acts as an inhibitor of stamen development in melon by playing a role in ethylene biosynthesis [42]. Through transcriptomics study, we had shown that AgNO_3_ spray resulted in inhibition of ethylene signalling in *C. grandis* [35]. In the present report, we observed that *Coccinia* homologs of many proteins involved in pollen development such as USP (UDP-sugar pyrophosphorylase), RPN10 (Regulatory particle non-ATPase 10), UGP2 (UTP-glucose-1-phosphate uridylyltransferase 2), EDA9 (Embryo sac Development Arrest 9), TKPR1 (Tetraketide alpha-pyrone reductase 1), C4H (Cinnamic acid 4-hydroxylase), TPLATE, CDC2 (Cell division control protein 2 homolog A), ANN5 (Annexin D5), RAB1C (Ras-related protein RABD2c) were enriched in Ag-H buds compared to female buds [59–69] (Fig. 5a & b). LAP5 (LESS ADHESIVE POLLEN 5), TKPR1 and TKPR2 (Tetraketide alpha-pyrone reductase 2), which are involved in sporopollenin biosynthesis and pollen exine formation were upregulated upon AgNO_3_ treatment (Fig. 5a, b) [63, 64, 70, 71]. Also, *Coccinia* homologs for OASA1 (O-acetylserine sulfhydrylase 1), AtkdsA1 (Putative aldolase-type TIM barrel family protein), TCTP (Translationally-controlled tumor protein), ACAT2 (Acetoacetyl-CoA thiolase 2), ANN5, RAB1C proteins involved in pollen germination and pollen tube growth were enriched in Ag-H buds compared to female buds (Fig. 5a & b) [68, 69, 72–74]. Although, these proteins were expressed upon AgNO_3_ treatment, the pollens of Ag-H buds failed to germinate in vitro as well as did not fertilize the female buds [18]. Interestingly, Ag^+^ ions were able to induce the development of stamens even in absence of Y-chromosome, which would otherwise get arrested in female plants [18]. We speculate that Y-chromosome may be essential for providing pollen fertility as the pollens from Ag-H flowers buds were sterile in nature.

### Pollen maturation in male, GyM-H and Ag-H buds

Comparison of protein accumulation profiles from male buds to those of GyM-H and Ag-H buds resulted in the identification of many proteins that were expressed at different levels in these flower types. *Coccinia* homologs of proteins such as LAP6 (LESS ADHESIVE POLLEN 6), CEP1 (KDEL-tailed cysteine endopeptidase), OASB (O-acetylserine sulfhydrylase B), VHA-A (V-type proton ATPase subunit a3) and PIRL9 (Plant intracellular Ras-group-related LRR protein 9) were enriched in male and GyM-H buds compared to Ag-H and female buds (Fig. 5a & b). LAP6 protein known to be involved in pollen exine formation/sporopollenin biosynthesis in *Arabidopsis*, was found to be enriched in male and GyM-H buds compared to Ag-H and female buds of *C. grandis* (Fig. 5a & b) [70, 71]. In contrast, LAP5, (also involved in pollen exine formation) was seen to be enriched in male and Ag-H buds but not in GyM-H and female buds (Fig. 5a & b) [70, 71]. A mutation in *Arabidopsis* CEP1, a KDEL-tailed cysteine endopeptidase, has been shown to exhibit aborted tapetal PCD and decreased pollen fertility with abnormal pollen exine [75]. On the other hand, VHA-A (V-type proton ATPase catalytic subunit A) protein is strictly required for proper development of male gametophyte [76]. Consistently, VHA-A had a high expression in male and GyM-H buds (Fig. 5a & b). Plant intracellular ras-group-related LRR protein (PIRL9) has been shown to play an important role in the process of microgametogenesis and pollen development [77]. PIRL9 had high expression in male and GyM flower buds (Fig. 5a & b). OASB (O-acetylserine sulfhydrylase) protein that has been known to be required for pollen tube elongation and fertilization in *Arabidopsis* showed high expression in male and GyM-H flower buds (Fig. 5v) [74].

Many proteins, however, were enriched specifically in male buds with very low levels in GyM-H, Ag-H and female buds. AMS (ABORTED MICROSPORES), UNE5 (UNFERTILIZED EMBRYO SAC 5) and iPGAM2 *Coccinia* homologs were enriched in middle-staged male buds (Fig. 5a & b). UNE5 is known to be important in the process of pollen tube development [78]. Mutation in transcription factor AMS (ABORTED MICROSPORES) results in defective tapetum development, abnormal microspores and non-viable pollens [79]. Similarly, *Coccinia* homologs ACOS5 (Acyl-CoA Synthetase) and QRT3 (QUARTET 3) involved in pollen exine formation were also enriched in middle-staged male buds (Fig. 5a & b) [64, 80, 81]. Another protein FUM1 (Fumarate hydratase 1), involved in pollen tube development was enriched in early-staged male buds compared to GyM-H and Ag-H buds (Fig. 5a & b) [78]. All these observations indicate that these proteins probably govern the pollen viability and lack of their expression in GyM-H and Ag-H buds leads to the sterile pollens.

## Conclusion

Current study has led to the identification of many proteins expressed in the flower buds of male, female and gynomonoecious forms of *Coccinia grandis* at early and middle stages of development. Protein search against the in silico translated *C. grandis* flower bud transcriptome database has resulted in the identification of 3385 proteins. Simultaneously, protein search analysis against cucumber protein sequences (Phytozome) led to 434 new proteins that could not be detected through *C. grandis* flower bud transcriptome. This has further improved the number of proteins identified from the *C. grandis* flower buds in the current study. Also, changes in the proteome profile upon AgNO_3_-mediated sex modification in female plants were revealed. Through protein interaction network maps, we observed that proteins involved in ethylene biosynthesis such as UBA2A, UBA2C and EFE were upregulated in female buds of *C. grandis* indicating high levels of ethylene similar to the female buds of monoecious cucurbits such as melon, cucumber and watermelon. This could suggest that the role of ethylene in stamen inhibition might be conserved not only in monoecious cucurbits but also in dioecious *C. grandis.* AgNO_3_ treatment was able to induce stamen development in female plants of *C. grandis*. However, the pollens from Ag-H and GyM-H buds were sterile in nature. There could be two possibilities to explain the cause of pollen sterility in GyM-H and Ag-H flower buds. The flower buds of gynomonoecious and male plants could differ in the timing or level of gene expression leading towards the incomplete development of pollens. Alternatively, genes governing pollen fertility might be Y-linked and absent from GyM-H and Ag-H forms. As till now, complete genome sequence of *Coccinia* is not available, it would be difficult to ascertain this. Future investigation is required to unravel the mechanistic link between ethylene mediated stamen inhibition as well as the role of Y-chromosome in pollen fertility. Overall, in light of the limited molecular resources for such non-model dioecious species, our current flower bud proteome datasets will serve as invaluable novel resources for future identification of key molecular players involved in the development of unisexual flowers in cucurbitaceae, the second largest horticultural plant family in terms of economic importance.

## Methods

### Flower bud collection

Clones of wild-type male, female and gynomonoecious (GyM, herbarium voucher: Tripura University Campus, Karmakar, 433) forms of *C. grandis* were grown in the experimental plot at IISER Pune, India [18]. Some of the female plants were chemically masculinized by a foliar spray of 35 mM AgNO_3_ solution to the basal leaves, leading to the development of morphologically hermaphrodite flowers (Ag-H). Both early and middle-staged flower buds of male, female, gynomonoecious and chemically masculinized female plants of *C. grandis* were harvested as per the prevoius protocol [35]. Six biological replicates for each of the samples were used in this study. Each biological replicate consisted of a pool of 4–5 flower buds of uniform size, crushed in liquid nitrogen and stored at − 80 °C until further use. Overall, experimental plan is depicted schematically in Fig. 1. Early-staged male flower buds chosen for this study showed the development of stamen intitials without any sign of carpel initials, while early-staged female flower buds (stages 3–4) had both carpel as well as stamen initials. We have noticed that stamen growth in female flower gets arrested around stages 4–5 [18]. The early-staged flower buds were chosen such that the stamen inhibition regulation can be studied [35]. However, in both types of hermaphrodite flowers (Ag-H and GyM-H), stamens and carpels develop simultaneously during early as well as middle stages of development. Pollen maturation process was also of interest since only male flowers produced viable pollens. Both types of hermaphrodite flowers (Ag-H and GyM-H) produced non-viable pollens [18]. Hence, middle-staged flower buds were chosen such that pollen maturation process can be investigated [35].

### Protein extraction

Protein extraction was carried out from flower buds of *C. grandis* as per Isaacson*,* et al. [82] with some modifications. Flower buds were crushed fine in liquid nitrogen and 50 mg of the powdered samples, weighed in 15 mL tubes (TARSONS) were used for each biological replicate. Five mL of chilled 10% TCA/Acetone with 2% β-mercaptoethanol was added to these tubes, vortexed and incubated for at least 4 h at − 20 °C. The tubes were further centrifuged at 10000 rpm for 20 mins and the supernatant was carefully discarded. The excess acetone in the pellet was evaporated in the fume-hood. Ten mg of insoluble PVPP (Polyvinylpolypyrrolidone) was added to this pellet followed by addition of 5 mL of extraction buffer containing 0.7 M sucrose, 0.1 M KCl, 0.5 M Tris-HCl, pH 7.5, 50 mM EDTA, 0.5% SDS and β-mercaptoethanol at a final concentration of 2% (v/v) and vortexed vigorously for 15 mins. The tubes were centrifuged for 20 mins at 4 °C and 10000 rpm. The supernatant was transferred to a fresh 15 mL tube to which 5 mL Tris equilibrated phenol (pH 8.0) was added and vortexed vigorously for 15 mins. Phase separation was carried out by centrifugation at 10000 rpm for 20 mins at 25 °C. The upper phenolic phase was transferred to a fresh tube and washed with the extraction buffer (without SDS). The process was repeated twice and the protein was precipitated using 3–5 volumes of chilled 0.1 M ammonium acetate in methanol containing 2% β-mercaptoethanol. Pelleting and washing of the protein was performed as described previously [82]. The dried protein pellet was reconstituted in 0.1% RapiGest SF (Waters) and quantitated using Bradford method (microtitre plate method, BioRad).

### In-solution digestion and peptide purification

For in-solution digestion of the extracted proteins, 100 μg of reconstituted proteins was taken in 1.5 mL LoBind tubes (Eppendorf) and diluted using 0.1% RapiGest SF (prepared in fresh 50 mM ammonium bicarbonate solution) to make a final volume of 100 μL. This protein solution was heated at 80 °C for 15 mins on thermomixer at 1250 rpm. Five μL of 100 mM DTT (Dithiothreitol, freshly prepared) was added to the preheated protein solution and incubated at 60 °C, 1250 rpm for 15 mins. The protein solution was cooled to room temperature and 5 μL of 200 mM iodoacetamide was added and incubated in dark for 30 mins at room temperature. Three μg of trypsin (Trypsin GOLD, Promega, MS-Grade) was added to the protein for digestion and incubated for 16 h at 37 °C and 1250 rpm. The reaction was stopped by adding 2 μL of formic acid (Sigma, MS-Grade) and incubated at 37 °C under static condition for 45 mins. The tubes were centrifuged at 15000 rpm for 30 mins at 4 °C and supernatant was collected in a fresh 1.5 mL LoBind tube and stored at − 80 °C until further use. The tryptic peptides were purified using C18 ZipTip (Milipore) by following manufacturer’s instructions. The peptide fractions were vacuum dried and reconstituted using 3% ACN with 0.1% formic acid to yield a final concentration of 1 μg/μL.

### MS data acquisition for IDA and SWATH

The reconstituted peptides were further diluted to yield concentrations of 3 μg/5 μL for IDA acquisitions and 1 μg/5 μL for SWATH acquisitions. Peptide digests were separated by using an Eksigent MicroLC 200 system equipped with Eksigent C18-reverse phase column (100*0.3 mm, 3 μm, 120 Å). Samples were analyzed on AB-SCIEX Triple TOF 5600 mass spectrometer. The LC separation methods and parameter settings for MS acquisitions have been followed as per the protocol of Korwar*,* et al. [83]. The mass spectrometry proteomics data have been deposited to the ProteomeXchange Consortium via the PRIDE [84] partner repository with the dataset identifier PXD011064.

Peptide spectral library and data base search - IDA mass spectrometric files were searched using ProteinPilot software, version 5.0.1.0.4874 (AB SCIEX) with the Paragon algorithm against in silico translated in-house *C. grandis* flower bud transcriptome at FDR (false discovery rate) ≤ 1%. The ProteinPilot output file (.group) was used as a standard peptide spectral library. In order to identify proteins that might have gone undetected due to the limitation of *Coccinia* transcriptome database coverage, IDA mass spectrometric files were also searched against *Cucumis sativus* protein sequences available in Phytozome [85]. SWATH analysis was performed for six biological replicates and technical triplicates each from early-staged male, female, Ag-H and GyM-H buds as well as middle-staged male, female, Ag-H and GyM-H buds. The spectral alignment and targeted data extraction of SWATH-MS data was performed using Peakview software, version 2.2 (AB SCIEX). The peptide data (.MRKVW) files were used for quantification of proteins using Markerview software, version 1.3 (AB SCIEX). Normalization was performed using β-galactosidase and actin-7 (TRINITY_DN116897) peak area followed by the total area sum. The peptides with a *P* ≤ 0.05 were considered for quantification. PCA-DA analysis was carried out using Markerview in order to study the relationship between all the samples and replicates followed by t-test to identify DEPs among each pairwise comparisons in which fold change was set as ≥1.5, and significance of *P* ≤ 0.05 was chosen as cut-off.

### Protein-protein interaction network analysis

Accession ID of *Arabidopsis thaliana* homologs of the proteins identified by proteomic analysis were used to study the protein-protein interaction using Cytoscape 3.6 [86]. The STRING database for *Arabidopsis thaliana* was used to import the annotation, interaction and further analysis was performed using STRING App 1.2.2 [87]. Gene enrichment analysis was performed and proteins grouped under reproductive process and floral development, were used for generating network representation.

### Transcript abundance analysis

In order to estimate the transcript levels for selected differentially enriched proteins, qRT-PCR was carried out using the same tissue samples that were used for proteomics. Complementary DNA (cDNA) synthesis was performed with two micrograms of total RNA by SuperScript IV reverse transcriptase (Invitrogen) using an oligo(dT) primer. BIO-RAD CFX96 machine was used for carrying out the qRT-PCR with gene-specific forward and reverse primers (Additional file 10: Table S1). Takara SYBR Premix Ex *Taq* II (Takara Bio Inc.) was used for the reactions and PCR plates were incubated at 95 °C for 3 min followed by 40 cycles of 95 °C for 15 s, 58 °C for 15 s and 72 °C for 15 s. Melting curve analysis was performed to ascertain PCR specificity. Data were analysed using the 2^–∆∆CT^ method and *CgACT2* was chosen as a reference gene for normalization [88].

## Additional files

Additional file 1: **Data S1.** Detailed report of protein identification from *C. grandis* flower buds using Paragon algorithm with ProteinPilot v5.0.1. (XLSX 12245 kb)

Additional file 2: **Data S2.** BLAST2GO annotation table for the *C. grandis* flower bud proteins detected in this study. (XLSX 640 kb)

Additional file 3: **Figure S1.** Enzyme code distribution analysis for the detected *C. grandis* flower bud proteins using BLAST2GO v5. (TIF 1214 kb)

Additional file 4: **Figure S2.** Heatmap depicting expression profiles of 2262 hierarchically clustered proteins from different stages of *C. grandis* flower bud samples. AGE (Red), Early-staged Ag-H; AGM (Green), Middle-staged Ag-H; FE (Blue), Early-staged Female; FM (Turquoise), Middle-staged Female; GE (Pink), Early-staged GyM-H; GM (Yellow), Middle-staged GyM-H; ME (Grey), Early-staged Male; MM (Black), Middle-staged Male. (TIF 56 kb)

Additional file 5: **Data S3.** Normalized peak areas for each of the quantified proteins across all the samples and pairwise differential expression analysis results with respective fold-changes as well as *P*-values. DEPs with fold change ≥1.5 and *P* ≤ 0.05 are shown in highlighted cells for all pairwise comparisons. (XLSX 7759 kb)

Additional file 6: **Figure S3.** Venn diagram showing the overlap of differentially expressed proteins between all the pairwise comparisons at early (A) and middle stages (B) of flower development. (PDF 343 kb)

Additional file 7: **Data S4.** Overlap of differentially expressed proteins between all the pairwise comparisons at early stage of flower development. (XLSX 42 kb)

Additional file 8: **Data S5.** Overlap of differentially expressed proteins between all the pairwise comparisons at middle stage of flower development. (XLSX 38 kb)

Additional file 9: **Data S6.** Normalized peak areas for the proteins involved in male and female reproductive organ development, seed development and embryo development along with their respective *Arabidopsis* accession IDs. (XLSX 144 kb)

Additional file 10: **Table S1.** Primers used for qRT-PCR in the current study. (PDF 39 kb)

## Data Availability

The mass spectrometry proteomics data have been deposited to the ProteomeXchange Consortium via the PRIDE partner repository with the dataset identifier PXD011064.

## Abbreviations

Ag-H flower buds: Hermaphrodite flowers from a plant treated with silver nitrate
GO: Gene Ontology
GyM: Gynomonoecious
GyM-H flower buds: Hermaphrodite flowers from a gynomonoecious plant
IDA: Information Dependent Acquisition
LC-MS: Liquid Chromatography–Mass Spectrometry
NGS: Next-Generation Sequencing
Nr: Non-redundant protein database
PCA-DA: Principal Component Analysis and Discriminant Analysis
qRT-PCR: Quantitative Real-Time PCR
SWATH-MS: Sequential Window Acquisition of All Theoretical Mass Spectra
