# Supplementary figures and images for: Flower bud proteome reveals modulation of sex-biased proteins potentially associated with sex expression and modification in dioecious Coccinia grandis
Source: BMC Plant Biol. 2019 Jul 23;19:330. doi: 10.1186/s12870-019-1937-1 (PMC6651928; doi:10.1186/s12870-019-1937-1)

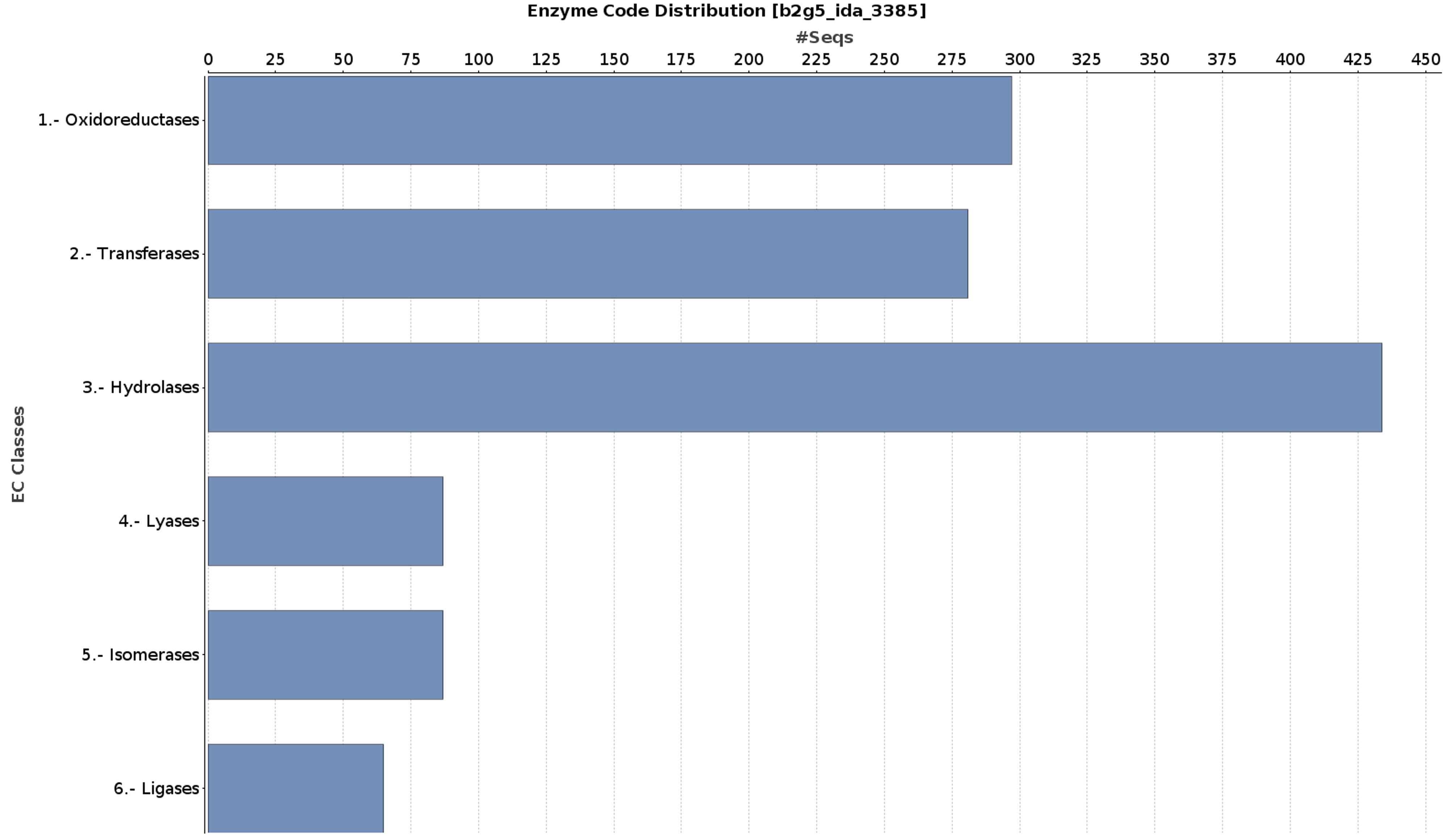

Supplement: Supplementary file 3 — Figure S1. Enzyme code distribution analysis for the detected C. grandis flower bud proteins using BLAST2GO v5. (TIF 1214 kb) [file 12870_2019_1937_MOESM3_ESM.tif]

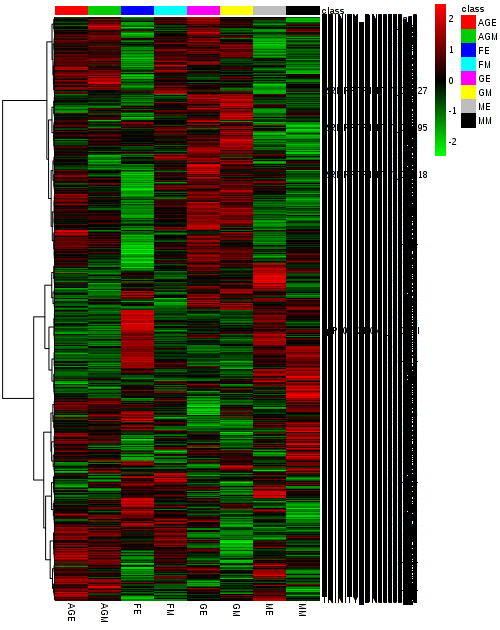

Supplement: Supplementary file 4 — Figure S2. Heatmap depicting expression profiles of 2262 hierarchically clustered proteins from different stages of C. grandis flower bud samples. AGE (Red), Early-staged Ag-H; AGM (Green), Middle-staged Ag-H; FE (Blue), Early-staged Female; FM (Turquoise), Middle-staged Female; GE (Pink), Early-staged GyM-H; GM (Yellow), Middle-staged GyM-H; ME (Grey), Early-staged Male; MM (Black), Middle-staged Male. (TIF 56 kb) [file 12870_2019_1937_MOESM4_ESM.tif]
