# Supplementary material for: Flower bud proteome reveals modulation of sex-biased proteins potentially associated with sex expression and modification in dioecious Coccinia grandis
Source: BMC Plant Biol. 2019 Jul 23;19:330. doi: 10.1186/s12870-019-1937-1 (PMC6651928; doi:10.1186/s12870-019-1937-1)
Supplement: Supplementary file 6 — Figure S3. Venn diagram showing the overlap of differentially expressed proteins between all the pairwise comparisons at early (A) and middle stages (B) of flower development. (PDF 343 kb) [file 12870_2019_1937_MOESM6_ESM.pdf]

A

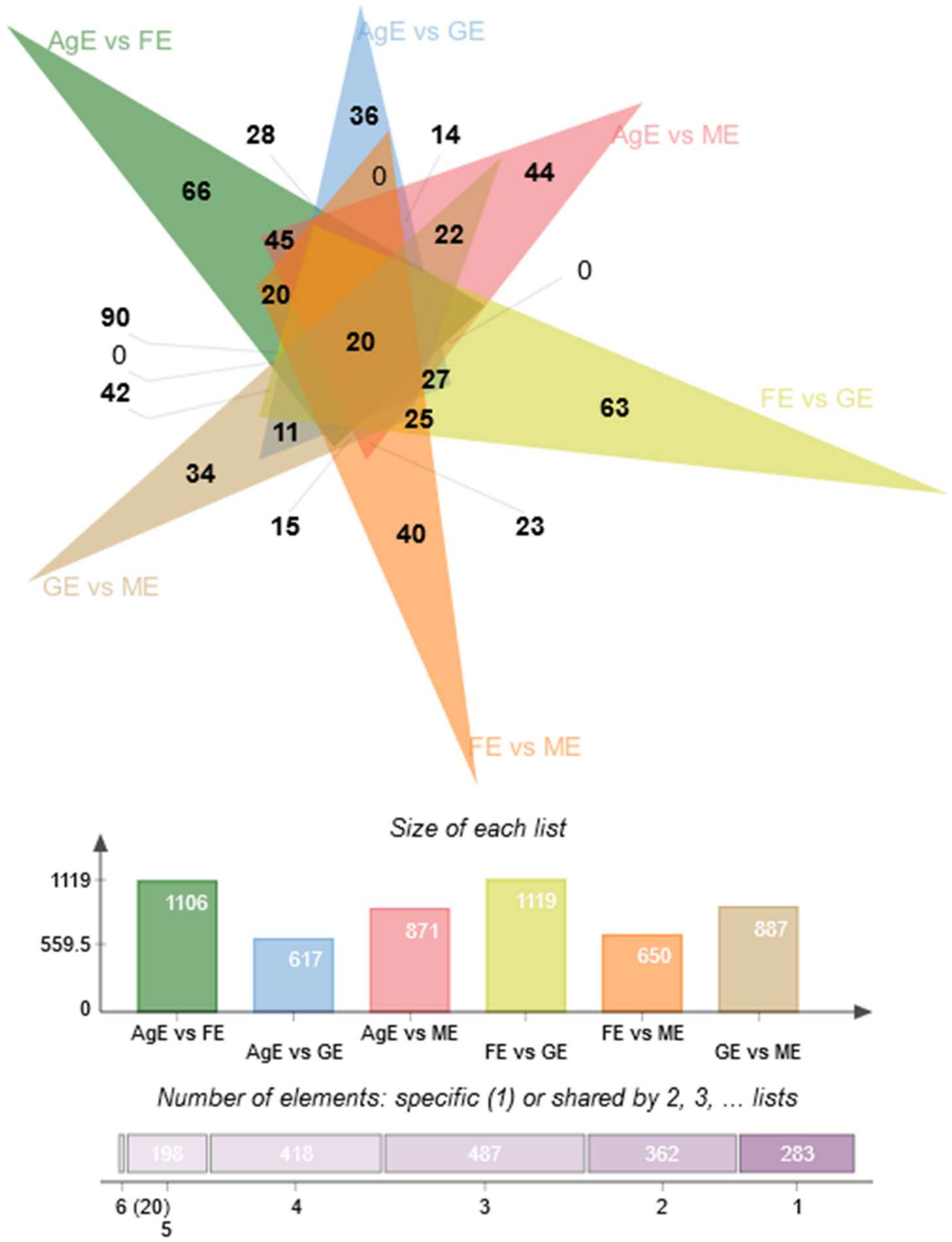

DEP Overlap between pairwise comparisons at early stage

B

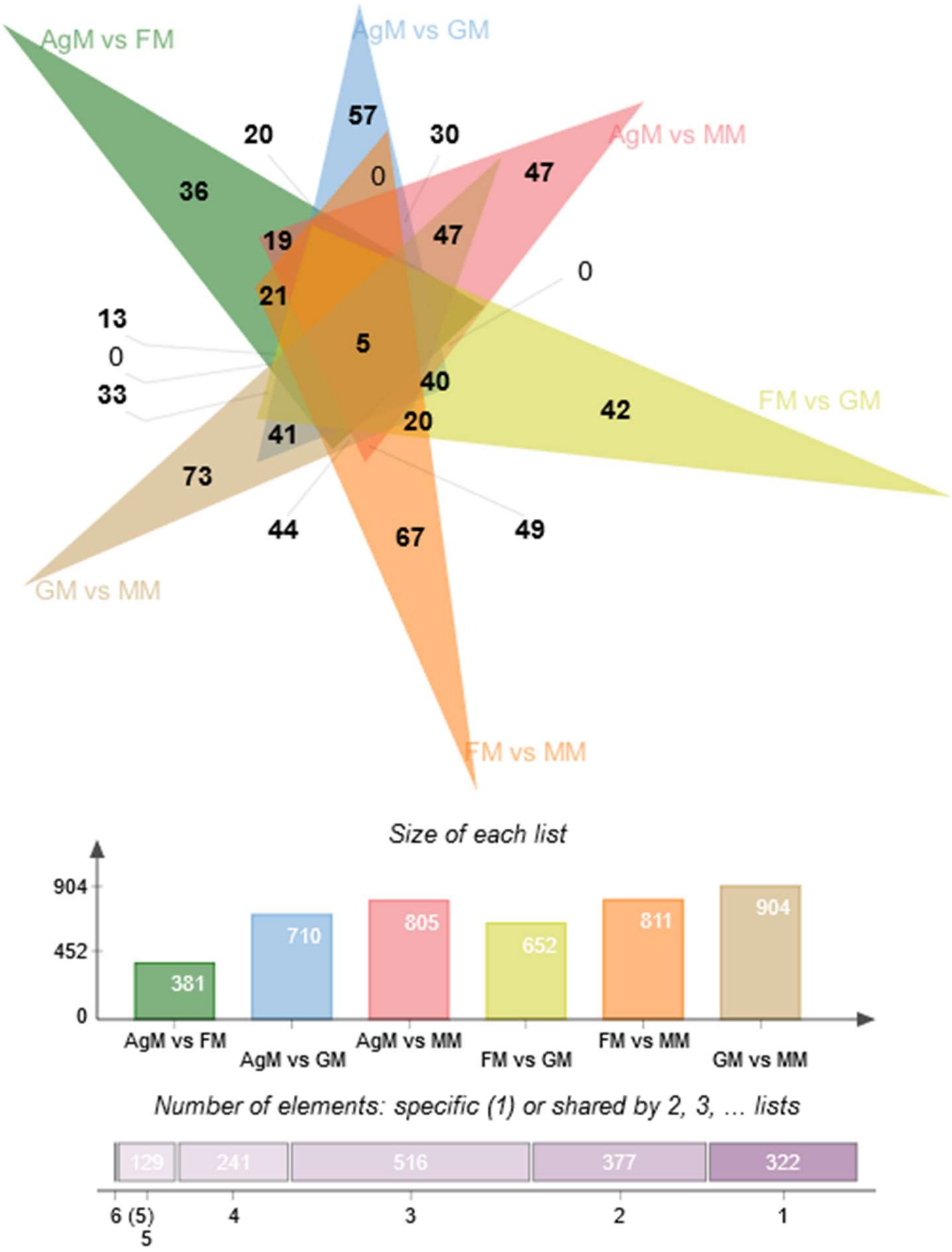

DEP Overlap between pairwise comparisons at middle stage
