# Supplementary material for: Flower bud proteome reveals modulation of sex-biased proteins potentially associated with sex expression and modification in dioecious Coccinia grandis
Source: BMC Plant Biol. 2019 Jul 23;19:330. doi: 10.1186/s12870-019-1937-1 (PMC6651928; doi:10.1186/s12870-019-1937-1)
Supplement: Supplementary file 10 — Table S1. Primers used for qRT-PCR in the current study. (PDF 39 kb) [file 12870_2019_1937_MOESM10_ESM.pdf]

Table S1. Primers used for qPCR in the current study

Primer Name	Sequence (5′ -> 3′)
EFE qF	CTTGGTGGAGAAAGAGGCGGAG
EFE qR	ACCTTGGCTCCTTCGCTTGAAA
AMS qF	CCGAACATCCGAAATCGACTGAAC
AMS qR	TAGTCAGTTTCGGTCGGTTTTTAGC
AIM1 qF	TCCGAAAATGCAGCGTCGAGAT
AIM1 qR	AGCAAACCATTTCAAAGCCAGCTC
PDIA6 qF	ATAAGTCGAGCTCCGCAGACTA
PDIA6 qR	TGTAAAGATCGGGAAATCGGGCT
TKPR1 qF	ACAAGGCCATCTTTCTTCACCTT
TKPR1 qR	AACTTCCACGTGTACGGTCGAG
ETHE1 qF	CCGTCGTCTTCCTCGTCTAAGC
ETHE1 qR	AAGCGCAGGTTTATCAGGGTGG
